# Two independent epigenetic biomarkers predict survival in neuroblastoma

**DOI:** 10.1186/s13148-015-0054-8

**Published:** 2015-02-27

**Authors:** Yania Yáñez, Elena Grau, Virginia C Rodríguez-Cortez, David Hervás, Enrique Vidal, Rosa Noguera, Miguel Hernández, Vanessa Segura, Adela Cañete, Ana Conesa, Jaime Font de Mora, Victoria Castel

**Affiliations:** Pediatric Oncology Unit, Hospital Universitari i Politècnic La Fe, Avda Fernando Abril Martorell, Valencia, 46026 Spain; Chromatin and Disease Group, Cancer Epigenetics and Biology Programme (PEBC) Bellvitge Biomedical Research Institute (IDIBELL), Gran Via de L’Hospitalet, Barcelona, 08908 Spain; Biostatistics Unit, Instituto de Investigación Sanitaria La Fe, Avda Fernando Abril Martorell, Valencia, 46026 Spain; Genomics of Gene Expression Lab, Centro de Investigaciones Príncipe Felipe, Carrer d’Eduardo Primo Yúfera, Valencia, 46012 Spain; Department of Pathology, Medical School, University of Valencia, Avda Blasco Ibáñez, Valencia, 46010 Spain; Department of Pathology, Hospital Universitari i Politècnic La Fe, Avda Fernando Abril Martorell, Valencia, 46026 Spain; Laboratory of Cellular and Molecular Biology, Instituto de Investigación Sanitaria La Fe, Avda Fernando Abril Martorell, Valencia, 46026 Spain

**Keywords:** Neuroblastoma, Epigenetic biomarkers, Promoter methylation

## Abstract

**Background:**

Neuroblastoma (NB) is the most common extracranial pediatric solid tumor with a highly variable clinical course, ranging from spontaneous regression to life-threatening disease. Survival rates for high-risk NB patients remain disappointingly low despite multimodal treatment. Thus, there is an urgent clinical need for additional biomarkers to improve risk stratification, treatment management, and survival rates in children with aggressive NB.

**Results:**

Using gene promoter methylation analysis in 48 neuroblastoma tumors with microarray technology, we found a strong association between survival and gene promoter hypermethylation (*P* = 0.036). Hypermethylation of 70 genes significantly differentiated high-risk survivor patients from those who died during follow-up time. Sixteen genes with relevant roles in cancer biology were further validated in an additional cohort of 83 neuroblastoma tumors by bisulfite pyrosequencing. High promoter methylation rates of these genes were found in patients with metastatic tumors (either stage metastatic (M) or metastatic special (MS)), 18 months or older at first diagnosis, *MYCN* amplification, relapsed, and dead. Notably, the degree of methylation of retinoblastoma 1 (*RB1*) and teratocarcinoma-derived growth factor 1 (*TDGF1*) predicts event-free and overall survival independently of the established risk factors. In addition, low *RB1* mRNA expression levels associate with poor prognosis suggesting that promoter methylation could contribute to the transcriptional silencing of this gene in NB.

**Conclusions:**

We found a new epigenetic signature predictive for NB patients’ outcome: the methylation status of *RB1* and *TDGF1* associate with poorer survival. This information is useful to assess prognosis and improve treatment selection.

**Electronic supplementary material:**

The online version of this article (doi:10.1186/s13148-015-0054-8) contains supplementary material, which is available to authorized users.

## Background

Neuroblastoma originates from the sympathico-adrenal lineage of the neural crest and is the most common extracranial solid tumor in early childhood. This tumor exhibits contrasting patterns of clinical behavior ranging from spontaneous remission to rapid tumor progression and death. Prognosis classically depends on age at diagnosis [1], tumor stage [2], *MYCN* oncogene amplification status [3,4], and histology in a lesser extent [5]. Several well-characterized genetic abnormalities associated with neuroblastoma (NB) have been used to predict outcome, for example, DNA content [6], gain of chromosome arm 17q [7], or deletion of chromosome arm 1p [8] and 11q [9,10]. However, current knowledge of the molecular features of NB is not sufficient to explain the observed clinical heterogeneity.

There is an important body of work on NB to find robust biomarkers that could help to improve the standard response criteria and, consequently, patients’ survival. In this regard, several mRNA and miRNA classifiers have been established [11-13]. A recent study from SIOPEN shows that levels of tyrosine hydroxylase (*TH*) and paired-like homeobox 2B (*PHOX2B*) or doublecortin (*DCX*) mRNA in peripheral blood and bone marrow at diagnosis are independent predictors of survival [14]. Notably, high levels of *TH* and *PHOX2B* mRNA in peripheral blood identify ultrahigh-risk NB patients. Despite these new discoveries, survival rates in children with high-risk NB remain disappointingly low.

Epigenetic modifications, particularly the methylation of the 5′ position of cytosines, within CpGs dinucleotides at gene promoter regions, are essential regulatory mechanisms for normal cell development and may modulate gene expression without altering the DNA sequence. In the last decade, DNA-methylation studies have focused on identifying epigenetically modified genes to further understand NB pathogenesis and to find prognostic methylation markers. In this regard, global methylation studies have demonstrated that a methylator phenotype, characterized by the methylation of multiple CpG islands, is a hallmark of NB with poor prognosis [15]. Several tumor suppressor genes such as caspase 8 (*CASP8*), Ras association (RalGDS/AF-6) domain family member 1 (*RASSF1A*), cycling D2 (*CCND2*), CD44, *O*-6-methylguanine-DNA methyltransferase (*MGMT*), and PYD and CARD domain containing (*TMS1*), have been shown to be silenced in NB by aberrant hypermethylation of their promoters [16-18]. In general, screening studies in NB showed that frequently methylated genes are related to apoptotic pathways as well as to cell cycle regulation.

Recent studies in oncology research have increased genome coverage allowing the identification of new epigenetic biomarkers. In NB, two recent studies have investigated DNA methylation patterns using genome-wide technologies [19,20]. However, the number of clinically relevant epigenetic biomarkers is still very low. In here, we used methylation microarrays to identify robust and independent epigenetic biomarkers in NB.

## Results

### Genome-wide promoter methylation screening

With the purpose of identifying DNA methylation biomarkers, we first analyzed 48 primary NB tumors using the Infinium HumanMethylation27 BeadChip microarray. Clinical, biological, and follow-up data are summarized in Table 1. Patients were classified following the International Neuroblastoma Risk Group Staging System (INRGSS) guidelines as 10 L1 (stage 1 localized); 16 L2 (stage 2 localized), 18 M (metastatic), and 4 MS (metastatic special). Twenty patients relapsed: 1 L1 presented a local relapse and is alive and disease free, 7 L2 (5 presented local relapses, 1 metastatic and 1 combined, 4 of them died of disease); 11 M stages (2 presented local relapses, 3 presented metastatic relapses and 6 combined relapses, all of them died of disease). One MS stage presented a metastatic relapse and died due to disease progression.

**Table 1:** **Clinical and biological data from the 48 patients included in the microarray analysis**

	**INRG staging system**				
**Characteristics**	**L1**	**L2**	**M**	**MS**	**Total**
Number of patients	10	16	18	4	48
Pre-treatment risk group					
Very low + low	10	2	0	3	15
Intermediate	0	10	2	0	12
High	0	4	16	1	21
Age at diagnostic in months					
Median	11.1	18.6	27.6	4	16.1
Range	1.5-22.7	4.8-109.3	6.6-79.8	1.9-6.7	1.5-109.3
Patients over 18 months at diagnostic	4	9	11	0	24
Sex					
Female	6	8	6	2	22
Male	4	8	12	2	26
Primary site					
Adrenal	1	5	9	3	18
Abdominal	4	5	2	0	11
Cervical	0	1	0	1	2
Thoracic	2	0	4	0	6
Cervical-thoracic	0	1	0	0	1
Thoracic-abdominal	1	1	0	0	2
Pelvic	1	0	0	0	1
Other sites	1	3	3	0	7
Protocol of treatment					
LNESG I	2	0	0	0	2
LNESG II	1	0	0	0	1
EUNS	0	2	0	0	2
INES	3	5	2	2	12
HR-NBL-1	0	4	9	0	13
Other (national protocols)	4	4	7	2	17
MYCN status					
Amplified (%)	0 (0%)	4 (25%)	8 (44%)	1 (25%)	13 (27%)
Not amplified (%)	10 (100%)	12 (75%)	10 (56%)	3 (75%)	35 (73)
1p status					
Normal (%)	8 (80%)	8 (50%)	8 (44%)	3 (75%)	27 (56%)
Deleted (%)	0 (0%)	4 (25%)	7 (39%)	0 (0%)	11 (23%)
Not determined (%)	2 (20%)	4 (25%)	3 (17%)	1 (25%)	10 (21%)
Patients with relapse (%)	1 (10%)	7 (44%)	11 (61%)	1 (25%)	20 (42%)
Type of relapse					
Local	1	5	2	0	8
Metastatic	0	1	3	1	5
Local + metastatic	0	1	6	0	7
Dead (%)	0 (0%)	5 (32%)	12 (67%)	1 (25%)	18 (38%)
Cause of death					
Disease progression	0	4	11	1	16
Other	0	1	1	0	2
Time of follow-up (month)					
Median	152.1	93.5	45.4	129.3	117.4
Range	87.8-208.3	9.4-206.5	3.4-161.2	17.2-214.9	3.4-214.9

**Figure 1:**
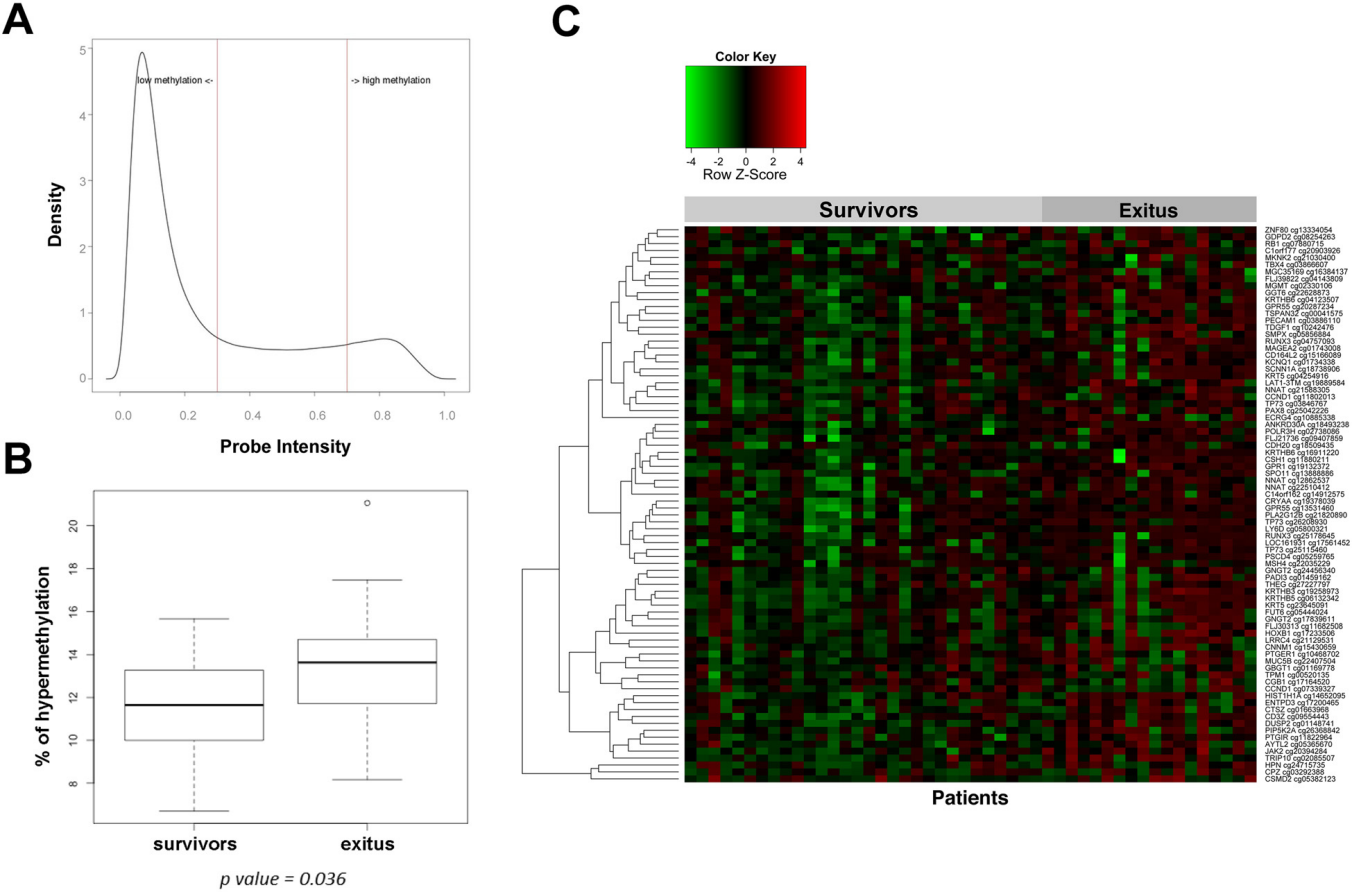
**DNA promoter methylation profiling from 48 NB primary tumors. (A)** Data distribution according to the probe intensity. **(B)** Association between gene promoter hypermethylation and patients’ status. **(C)** Heat map representation of DNA promoter methylation data of the 70 genes differentially methylated in the cohort of 48 NB patients.

Data distribution according to the methylation probe intensity from the Illumina array was shown to be bimodal (Figure 1A). Using minAS, a method for feature selection in multivariate data, cutoff values for data discretization were defined as follows: ≤0.3 hypomethylated ‘0’, >0.3 < 0.7 intermediate ‘0.5’, and >0.7 hypermethylated ‘1’.

In order to understand to which extent gene promoter methylation is relevant to NB, we analyzed differences in the methylation levels among different patient subgroups. The most striking result of this analysis revealed a significant association between poor survival and global gene promoter hypermethylation (*P* = 0.036) (Figure 1B). Patients who died during follow-up time had higher promoter hypermethylation rates than survivors (median follow-up time for survivors of 12 years). No association was detected between survival and global hypomethylation of gene promoters. Other types of subgrouping (by age at diagnosis, *MYCN* status, or relapse) did not reveal any significant changes. However, comparing patients at different NB stages (L1 and MS vs. L2 vs. M) did indicate a near-significant hypermethylation of the runt-related transcription factor 3 (*RUNX3*) gene (*P* = 0.05). These results suggest that the overall promoter hypermethylation status at diagnosis could predict survival and that NB stages can be associated with methylation changes at specific genes.

After establishing a significant association between promoter hypermethylation and NB, we then aimed to identify particular methylation events. Comparison of methylation values of high-risk patients who died of disease, and of high-risk patients who survived, revealed 70 genes that significantly differentiate these two groups (Figure 1C and Table 2). Interestingly, only specific CpG sites within promoter regions were methylated (compare total probes vs. significative probes that were methylated on Table 2 and see the scheme on Additional file 1). This result revealed the importance of sufficient CpG probes at promoter regions to capture the dynamics of methylation changes. Sixteen genes with high impact on cancer biology were selected for validation. The methylated probes and *P* values as well as the methylation level of the selected hypermethylated genes are shown in Table 3. Detailed information of these genes’ promoter methylation sites in each patient is described in Additional file 2. These genes regulate the following: maturation and maintenance of the overall structure of the nervous system (neuronatin (*NNAT*)); cell cycle progression (cyclin D1 (*CCND1*), janus kinase 2 (*JAK2*), retinoblastoma 1 (*RB1*), leucine-rich repeat C4 protein (*LRRC4*), and tumor protein P73 (*TP73*)); cell growth and differentiation (dual specificity phosphatase 2 (*DUSP2*), paired box 8 (*PAX8*), and hepsin (*HPN*)); tumorigenesis (melanoma antigen family A, 2 (*MAGEA2*), *RUNX3*, cathepsin Z (*CTSZ*), teratocarcinoma-derived growth factor 1 (*TDGF1*), and tetraspanin 32 (*TSPAN32*)); apoptosis (*JAK2* and esophageal cancer-related gene 4 protein (*ECRG4*)) and DNA repair mechanisms (*MGMT*).

**Table 2:** **Seventy significantly hypermethylated genes in high-risk NB patients**

**Genes**	**Total probes**	**Sig. probes**	**ID. Sig. probes**	***P*** **values**
*ANKRD30A*	2	1	cg18493238	0.044554738
*AYTL2*	2	1	cg05365670	0.035713814
*C14orf162*	2	1	cg14912575	0.005363655
*C1orf177*	2	1	cg20903926	0.012915253
*CCND1*	18	2	cg11802013, cg07339327	0.0341943541, 0.047611226
*CD164L2*	2	1	cg15166089	0.021147357
*CD3Z*	1	1	cg09554443	0.018393493
*CDH20*	1	1	cg18509435	0.047064214
*CGB1*	2	1	cg17164520	0.020905647
*CNNM1*	2	1	cg15430659	0.020905647
*CPZ*	2	1	cg03292388	0.022686711
*CRYAA*	2	1	cg19378039	0.004448711
*CSH1*	2	1	cg11880211	0.035724213
*CSMD2*	2	1	cg05382123	0.035713814
*CTSZ*	7	1	cg01663968	0.037116693
*DUSP2*	2	1	cg01148741	0.012020747
*ECRG4*	2	1	cg10885338	0.004398444
*ENTPD3*	2	1	cg17200465	0.022686711
*FLJ21736*	2	1	cg09407859	0.039315449
*FLJ30313*	2	1	cg11682508	0.035713814
*FLJ39822*	2	1	cg04143809	0.047611226
*FUT6*	2	1	cg05444024	0.03549584
*GBGT1*	2	1	cg01169778	0.037116693
*GDPD2*	2	1	cg08254263	0.034194354
*GGT6*	2	1	cg22628873	0.010030882
*GNGT2*	2	2	cg17839611, cg24456340	0.0135039944, 0.0183934926
*GPR1*	1	1	cg19132372	0.047064214
*GPR55*	2	2	cg20287234, cg13531460	0.005474794, 0.0343533092
*HIST1H1A*	2	1	cg14652095	0.021147357
*HOXB1*	2	1	cg17233506	0.020905647
*HPN*	2	1	cg24715735	0.022686711
*JAK2*	1	1	cg20394284	0.035713814
*KCNQ1*	23	1	cg01734338	0.034194354
*KRT5*	2	2	cg23645091, cg04254916	0.0067809343, 0.0072736595
*KRTHB3*	2	1	cg19258973	0.012020747
*KRTHB5*	2	1	cg06132342	0.000549806
*KRTHB6*	2	2	cg04123507, cg16911220	0.000926591, 0.0053636552
*LAT1-3TM*	1	1	cg19889584	0.013503994
*LOC161931*	2	1	cg17561452	0.044554738
*LRRC4*	2	1	cg21129531	0.013503994
*LY6D*	2	1	cg05800321	0.032446014
*MAGEA2*	2	1	cg01743008	0.034353309
*MGC35169*	2	1	cg16384137	0.021877651
*MGMT*	26	1	cg02330106	0.031115525
*MKNK2*	2	1	cg21030400	0.031115525
*MSH4*	2	1	cg22035229	0.044554738
*MUC5B*	2	1	cg22407504	0.030056033
*NNAT*	7	3	cg22510412, cg12862537, cg21588305	0.0182304654, 0.0211473571, 0.0324460138
*PADI3*	2	1	cg01459162	0.010896145
*PAX8*	2	1	cg25042226	0.007819456
*PECAM1*	2	1	cg03886110	0.021147357
*PIP5K2A*	2	1	cg26368842	0.047611226
*PLA2G12B*	2	1	cg21820890	0.015211786
*POLR3H*	1	1	cg02738086	0.044554738
*PSCD4*	2	1	cg05259765	0.012683212
*PTGER1*	2	1	cg10468702	0.022686711
*PTGIR*	2	1	cg11822964	0.035713814
*RB1*	21	1	cg07880715	0.047611226
*RUNX3*	19	2	cg04757093, cg25178645	0.0004487598, 0.0390263555
*SCNN1A*	2	1	cg18738906	0.044000585
*SMPX*	2	1	cg05856884	0.035713814
*SPO11*	2	1	cg13888886	0.001023813
*TBX4*	2	1	cg03866607	0.006780934
*TDGF1*	2	1	cg10242476	0.037116693
*THEG*	1	1	cg27227797	0.030056033
*TP73*	12	3	cg03846767, cg26208930, cg25115460	0.0038887033, 0.0324460138, 0.0470642136
*TPM1*	2	1	cg00520135	0.007819456
*TRIP10*	2	1	cg02085507	0.037116693
*TSPAN32*	2	1	cg00041575	0.007819456
*ZNF80*	2	1	cg13334054	0.012599325

**Table 3:** **Hypermethylated genes selected for pyrosequencing validation**

**Genes**	**Sign. probes**	**Total probes**	***P*** **values**	**Gene function**
*NNAT*	3	7	0.018, 0.021, 0.032	Involved in the maturation or maintenance of the overall structure of the nervous system
*TP73*	3	12	0.004, 0.032, 0.047	Participates in the apoptotic response to DNA damage
*CCND1*	2	18	0.034, 0.048	Essential for the control of the cell cycle at the G1/S (start) transition
*RUNX3*	2	19	0.0004, 0.039	Tumor suppressor gene
*CTSZ*	1	7	0.037	May be involved in tumorigenesis and metastasis
*DUSP2*	1	2	0.012	Regulates cellular proliferation and differentiation
*HPN*	1	2	0.023	Plays an essential role in cell growth and maintenance of cell morphology
*JAK2*	1	1	0.036	Involved in cell cycle progression, apoptosis, mitotic recombination, genetic instability, and histone modifications
*LRRC4*	1	2	0.014	Significantly downregulated in primary brain tumors. The exact function of the protein encoded is unknown
*MAGEA2*	1	2	0.034	May play a role in embryonal development and tumor transformation or aspects of tumor progression
*MGMT*	1	26	0.031	Involved in DNA repair mechanisms
*PAX8*	1	2	0.008	Transcription factor. Mutations in this gene are associated with carcinogenesis
*ECRG4*	1	2	0.004	Antiapoptotic gene
*RB1*	1	21	0.048	Negative regulator of the cell cycle
*TDGF1*	1	2	0.037	Plays an essential role in embryonic development and tumor growth
*TSPAN32*	1	2	0.008	Is one of several tumor-suppressing subtransferable fragments located in the imprinted gene domain of chromosome 11p15.5

### Validation of the prognostic power of DNA methylation biomarkers by bisulfite pyrosequencing

To validate the specific CpG methylated sites, we performed sequencing analysis of the 16 promoter genes. Primers for bisulfite pyrosequencing were carefully designed flanking the methylated CpG sites detected in the array. Validation of results was carried out with an independent cohort of 83 NB including 12 L1, 21 L2, 42 M, and 8 MS stages. Clinical and biological data from the validation cohort is summarized in Table 4.

**Table 4:** **Clinical and biological characteristics of the NB cohort used for pyrosequencing validation**

	**INRG staging system**				
**Characteristics**	**L1**	**L2**	**M**	**MS**	**Total**
Number of patients	12	21	42	8	83
Patients over 18 months at diagnostic	3	8	38	0	49
Sex					
Female	8	6	16	3	33
Male	4	15	26	5	50
MYCN status					
Amplified (%)	0	8 (38%)	16 (38%)	1 (12.5%)	25 (30%)
Not amplified (%)	12 (100%)	13 (62%)	23 (55%)	7 (87.5%)	55 (66%)
Not determined (%)	0	0	3 (7%)	0	3 (4%)
Patients with relapse (%)	3 (25%)	5 (24%)	22 (52%)	0	30 (36%)
Dead (%)	0	6 (29%)	25 (60%)	0	31 (37%)

**Figure 2:**
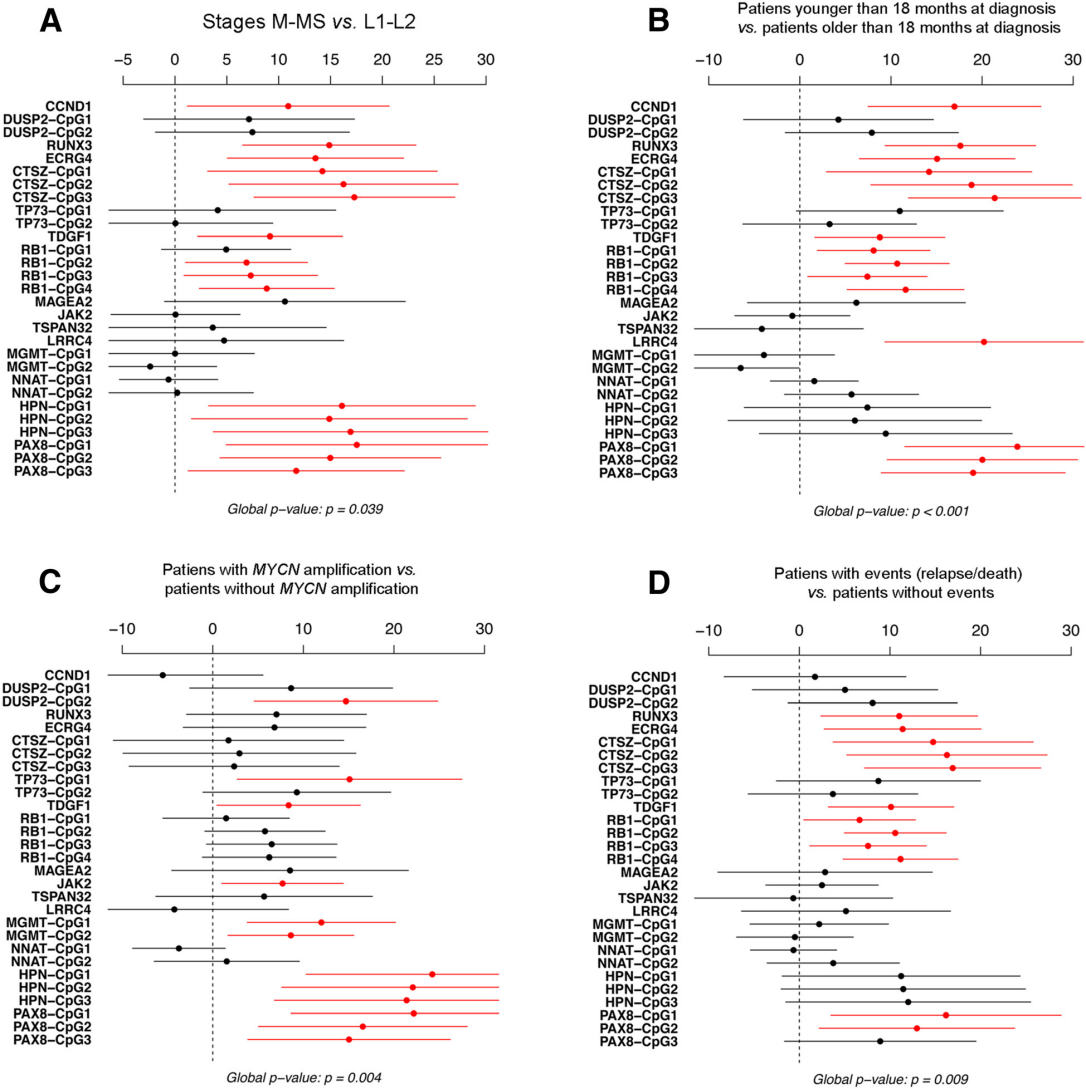
**Difference in methylation status of indicated genes (% units) among clinical variables in NB patients.** Global *P* value tests the hypothesis of equal methylation status in both groups. **(A)** Stages M - MS vs. L1-L2. **(B)** Patients younger than 18 months at diagnosis vs. patients older than 18 months at diagnosis. **(C)** MYCN-amplified patients vs. patients with no-MYCN amplification. **(D)** Patients with events (relapse/death) vs. patients without events.

We performed a multivariate analysis of variance (MANOVA) statistical analysis in order to study the methylation variations of selected genes among the established subgroups (see below and “Methods” section) of NB patients. Overall, we found significantly higher gene promoter methylation rates in patients with the following characteristics: metastatic tumors (either stage M or MS) (Figure 2A), aged 18 months or older at first diagnosis (Figure 2B) and *MYCN* amplification (Figure 2C). The same applies to patients who relapsed or died (Figure 2D). In particular, methylated status of *TDGF1* and *PAX8* allowed us to differentiate all the clinical subgroups mentioned above (Figure 2). In addition, high methylation rates of *RUNX3*, *ECRG4*, *CTSZ*, and *RB1* also distinguished all NB subgroups but not *MYCN*-amplified status (Figure 2). We also found highest methylation rates of *LRRC4* and *CCND1* in patients older than 18 months (Figure 2B). Furthermore, *DUSP2*, *TP73*, *JAK2*, *MGMT*, and *HPN* methylation rates were significantly higher in *MYCN*-amplified patients than in non-amplified patients (Figure 2C). These results demonstrate that these set of methylated gene promoters allow the discrimination of specific NB subgroups defined in Figure 2.

To study the influence of the degree of the 16 validated genes’ promoter methylation on survival, we evaluated them as independent prognostic variables by the elastic net penalized Cox’s regression model. Higher gene promoter methylation rates of *RB1*, *PAX8*, and *TDGF1* remained as independent predictors of overall survival (OS) after adjusting for known prognostic factors (Table 5A). On the other hand, *RB1* and *TDGF1* but not *PAX8* predicted worse event-free survival (EFS) (Table 5B). Information of *TDGF1* and *RB1* promoter methylation sites in the validation cohort of patients is described in Additional file 3. These regressions also confirmed the well-established stage, *MYCN* status, and age as independent predictors, thus further supporting the consistency of this model. The penalized coefficient of each independent variable directly associates with survival. Thus, *MYCN* amplification and staging have the highest influence on survival followed by the degree of *TDGF1* and *RB1* promoter methylation. Age at diagnosis had a lower impact in EFS and none in OS.

**Table 5:** **Cox elastic net results for (A) OS (B) and EFS**

**Variable**	**Penalized coefficient**
A. OS	
MYCN amplification	0.7217
Stage M	0.4871
*TDGF1*	0.0133
*RB1*	0.0045
*PAX8*	0.0002
B. EFS	
MYCN amplification	0.455
Stage MS	−0.34
Stage M	0.569
Age	0.002
*TDGF1*	0.009
*RB1*	0.005

Non-zero coefficients after elastic net penalization. Negative coefficients stand for variables lowering risk and positive coefficients for variables increasing risk.

### *RB1* expression in NB correlates with survival

**Figure 3:**
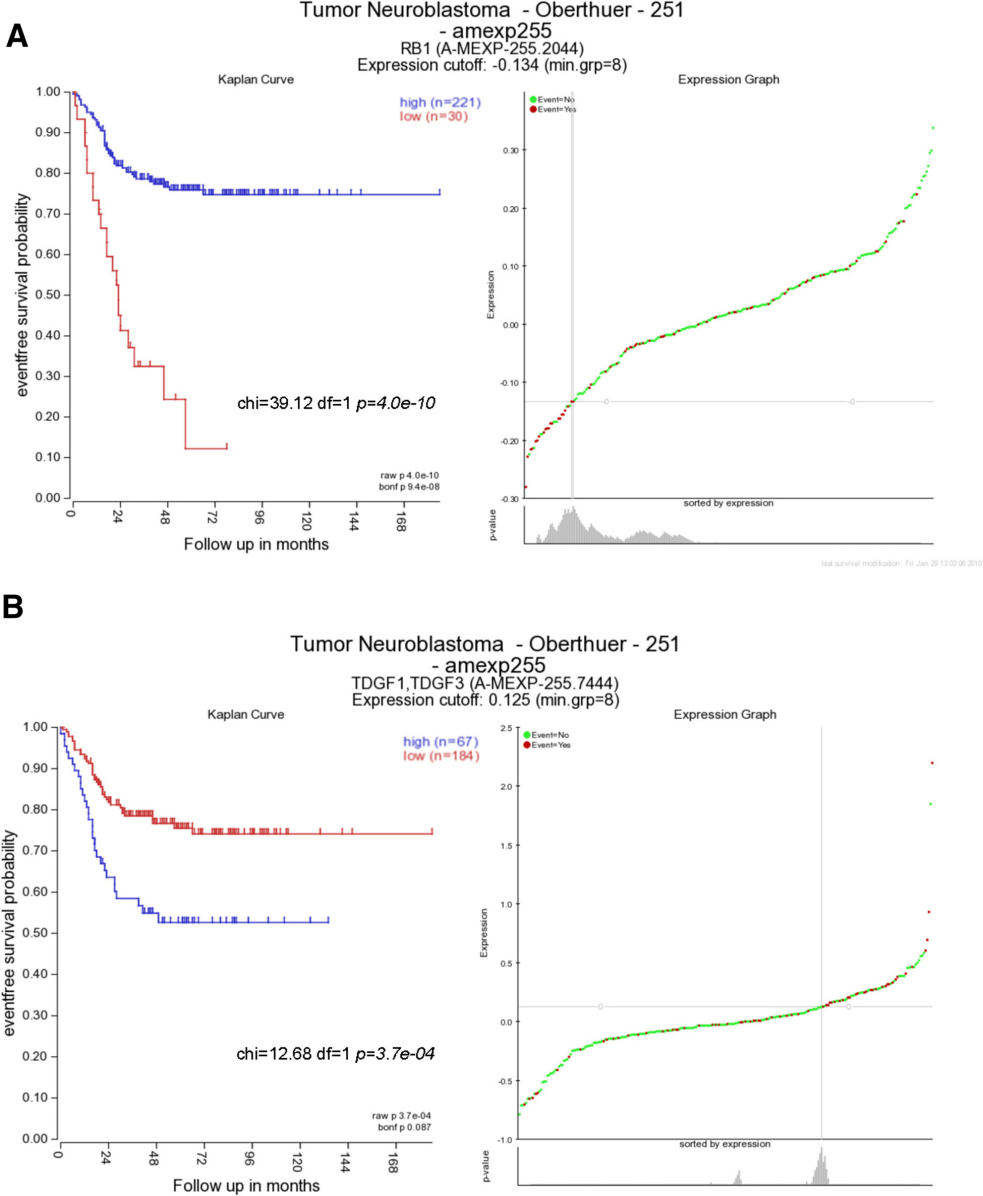
**Results of the analysis of a new cohort of 251 NB patients using the R2.** Expression graphs (on the right) and Kaplan-Meier plots (on the left) for RB1 **(A)** and TDGF1 **(B)** obtained using the R2: microarray analysis and visualization platform in a cohort of 251 NB patients.

Taking into account that *RB1* and *TDGF1* promoter methylation is one of the mechanisms responsible for the downregulation of these genes in other tumors [21,22], we explored *RB1* and *TDGF1* expression in NB, the two only independent predictors of EFS and OS that we found in this study. For this purpose, we analyzed a new cohort of 251 NB patients using the R2: microarray analysis and visualization platform (http://r2.arnc.nl). The results of this analysis are displayed in Figure 3. The Kaplan-Meier plots show the significant association between low expression levels of *RB1* and poorer outcome (Figure 3A). Based on these findings, promoter methylation could contribute to the transcriptional silencing of *RB1* in NB. On the other hand, low expression levels of *TDGF1* associates with better outcome in this patient cohort (Figure 3B). Since our results show that high methylation levels of *TDGF1* negatively affect patient survival, the *TDGF1* expression-Kaplan-Meier plots suggest that this particular epigenetic event on cg10242476 is not involved in *TDGF1* downregulation but rather in its expression. In order to investigate the positive correlation between CpG methylation and *TDGF1* expression, we explored the correlation between *TDGF1* expression and the DNA methyltransferases *DNMT1*, *DNMT3A*, and *DNMT3B*. As shown in Additional file 4, we found a significant correlation between *TDGF1* and *DNMT1* and *DNMT3B* but not between *TDGF1* and *DNMT3A* in the cohort of patients with *MYCN* amplification. These correlations further support that DNA methylation might as well be implicated in the upregulation of *TDGF1* expression.

## Discussion

Most of the DNA methylation studies in NB are experimentally limited because they used previously selected candidate genes based on their implication in cancer development or tumor biology [16,23-25]. A recent report described the signature of hypomethylated regions within non-promoter regulatory sites [20], thus complementing our findings on hypermethylated promoter regions. These characteristic profiles may reflect specific chromatin remodeling events that could contribute to the generation of chromosomal instability. Another recent genome-wide study has proposed new epigenetic biomarkers of interest in NB patients [19,26]. However, authors only used cell lines in the selection phase of potential prognostic DNA methylation biomarkers. Although candidate genes were further validated in 89 primary tumor samples, this approach may have skipped many candidate biomarkers occurring on primary tumors due to the passage-dependent epigenetic changes on cell lines. Lau et al. applied a genome-wide screen of DNA methylation changes using NB primary tumors [26]. In their study, they pre-selected candidate genes which may resulted in the loss of novel epigenetic biomarkers. In here, we adopted a non-targeted approach based on genome-wide screen of DNA-methylation changes. Our results reveal 70 candidate genes that showed epigenetic changes within the high-risk group. We further validated 16 out of 70 candidate genes, leaving for future validation studies the remaining group.

The two patient cohorts used in this study included a representative distribution of all the INRG-based NB subgroups, being among the highest and well-characterized NB cohorts used for genome-wide epigenetic studies so far reported to our knowledge. Interestingly, the degree of methylation of the proposed biomarkers is able to distinguish between different subgroups of NB: patients who were older than 18 months at diagnosis from younger than 18 months at diagnosis, patients with *MYCN*-amplified tumors from *MYCN* non-amplified tumors, patients with metastatic tumors from localized tumors and relapsed or dead patients from relapse-free survivors. These findings support the idea that aberrant DNA methylation could be related to NB pathogenesis. Notably, only *RB1* and *TDGF1* remained as independent prognostic predictors of poorer OS and EFS. Therefore, our predictive epigenetic biomarkers constitute a new set of robust risk predictors of the disease.

*TDGF1* promoter is hypomethylated and highly expressed in human-induced pluripotent stem (iPS) and embryonic stem (ES) cells [27]. Using the same Illumina array, our results reveal a different CpG site not only localized downstream but also within the first exon (5′-UTR) of *TDGF1* gene (cg10242476) that significantly predicts EFS and OS in NB patients. Importantly, *TDGF1* has been shown to be regulated by the ES cell-related transcription factors Oct4/Nanog and to a lesser extent by the DNA methylation status of the promoter region [28]. *TDGF1* has been found overexpressed in a variety of human tumors such as breast, colorectal, and gastric cancers, and high expression levels of this gene are associated with poorer prognosis in those tumors [29]. Using the microarray analysis and visualization platform R2 (http://r2.arnc.nl), we confirmed that high *TDGF1* expression levels are also associated with poor prognosis in NB (Figure 3B). Following this reasoning, higher methylation of this CpG would correlate with higher *TDGF1* expression and, therefore, with poorer prognosis. Interestingly, this CpG site is localized in the first exon of *TDGF1* transcript variant 1 (NM_003212) and coincides with the first intron of *TDGF1* transcript variant 2 (NM_001174136). One plausible explanation for this correlation is that intronic CpG methylation results in *TDGF1* transcript variant 2 expression. In this regard, regulation of imprinted *IGF2R* expression is mediated by methylation of an intronic CpG island [30]. Taken all together, these results suggest that intronic CpG (cg10242476) methylation in *TDGF1* transcript variant 2 positively regulates its expression in NB whereas CpG (cg27371741) hypomethylation in the first exon of *TDGF1* transcript variant 1 regulates its expression in embryonic stem cells. The strong association between hypermethylation and poor prognosis reflects the consequences of epigenetic changes occurring in high-risk NB.

Loss of *RB1* expression is associated with a higher grade of malignancies and seems to be a prognostic indicator in a variety of human tumors [31,32]. Hypermethylation of *RB1* CpG island is a common epigenetic event associated with the development of malignant nervous system tumors [22]. A clear correlation between loss of *RB1* expression and promoter hypermethylation was found in glioblastomas [32]. Although hypermethylation of *RB1* promoter has previously been reported in NB, no association with patient survival was included in the study [33]. Moreover, the methylation-specific PCR approach used to detect *RB1* promoter methylation does not allow to study the broad CpG sites analyzed with a genome-wide approach. We describe for the first time that the degree of *RB1* promoter methylation associates with poorer prognosis in NB patients. In agreement with our findings, using the publicly available R2 platform, we found that low expression levels of *RB1* associates with poorer outcome. These results suggest that *RB1* promoter methylation could contribute to its silencing and enhance NB development and aggressiveness.

## Conclusions

Biomarkers are playing an increasing role in the management of NB patients and, together with drug targets, represent the future analytical platforms for personalized clinical intervention. In this work, we demonstrate that high promoter methylation rates of *TDGF1* and *RB1* genes are independent predictive biomarkers of NB aggressiveness and disease progression. Our findings highlight the use of methylation profiling to identify risk-independent prognostic markers in NB and reinforce the connection between epigenetic events and NB biology. Taking into account that survival rates remain sadly low in high-risk NB patients, our epigenetic biomarkers are valuable tools for future patient stratification and treatment management.

## Methods

### Patients and samples

Tumor samples were resected from 131 children diagnosed with NB between years 1996 and 2010 in Spanish cooperative hospitals. Patients were included in different national and European studies (LNESG I and II, INES, EUNS, N-AR-99, N-II-92, and HR-NBL1) and carefully selected in order to have all NB subtypes represented (Table 1). Forty-eight NB tumor samples were used for genome-wide promoter methylation analysis, and an additional cohort of 83 tumor samples were used for validation. Staging and risk stratification was established according to International Neuroblastoma Risk Group (INRG) criteria [2]. However, very low- and low-risk patients were joined for the statistical analysis and were considered as low-risk group. Samples were centrally reviewed and classified according to the International Neuroblastoma Pathology Committee (INPC) system [5,34]. Biological studies included status of *MYCN* and 1p, both studied by FISH according to ENQUA guidelines [35,36]. Parents or guardians signed an informed consent statement for sample and data management. The study was approved by the Hospital La Fe Research Institute Ethical Committee.

### Genome-wide promoter methylation profiling

Genomic DNA was extracted from frozen tumor tissues by a standard proteinase K and phenol-chloroform extraction protocol. The quality and quantity of the extracted DNA was measured by A260 spectrophotometric absorbance. Genomic DNA bisulphite modification was carried out following the manufacturer’s instructions (Zymo Research). Promoter methylation analysis was performed using the Infinium HumanMethylation27 BeadChip (Illumina Inc., CA, USA) at the Spanish National Cancer Center (CEGEN-CNIO, Madrid, Spain). The Infinium HumanMethylation27 BeadChip allowed us to interrogate 27,578 highly informative CpG sites per sample, located within the proximal gene promoter regions of transcription. A file containing all CpG sites used in the Illumina array HumanMethylation27 BeadChip can be localized at http://support.illumina.com/array/array_kits/infinium_humanmethylation27_beadchip_kit/downloads.html. BeadStudio software (version 3, Illumina Inc, USA) [37,38] was used to analyze the data. For each CpG site, we calculated the beta-value (*b*-value), a quantitative measure of DNA methylation levels ranging from 0 for completely unmethylated to 1 for completely methylated cytosines.

### Bisulfite pyrosequencing

Promoter methylation data from the array was validated by bisulfite pyrosequencing. Genomic DNA was bisulfite modified using EZ DNA Methylation Gold Kit™ (Zymo Research). A subsequent PCR amplification was performed using biotinylated primers designed with the PyroMark Assay Design 2.0 software, Qiagen (Table 6). The pyrosequencing and data analysis were performed in a PyroMark Q24 System version 2.0.6 (Qiagen) following the manufacturer’s instructions.

**Table 6:** **Sequences of primers used for pyrosequencing validation**

**Genes**	**Amplification primers**		**Sequencing primers**
	**Name**	**Sequence**	
*CTSZ*	Forward	GTTGGGGYGTAGGTGGGTAT	GTAGTTTTGGGGGGA
	Reverse	[Btn]CACAAACATCAAAACTCACCCTAAATAT	
*DUSP2*	Forward	TTGAGTGGTTTGGGATAGGTTAA	GGGATAGGTTAAAGGGT
	Reverse	[Btn]AAAACRCAATCTAAACTAACCTAAAAACTT	
*CCND1*	Forward	[Btn]GGGYGGTTGGGTTTGTGTATTT	CATATTTATCTTTTTATCTTCTACT
	Reverse	ACCACCCTACCCTAATTCT	
*ECRG4*	Forward	ATTTTGGGTAAGGAGGGTTAG	GGGTAAAAGGGTTGT
	Reverse	[Btn]TACCATTTACCTCCTCTAAATTACCA	
*RUNX3*	Forward	[Btn]GGTTTTGGGAATTAGAGTTTAAGG	AAAAAAAAATCAATTCCAACT
	Reverse	ACTAACATAACCCCRAAATAATACATCCTA	
*TP73*	Forward	AGTTAGTTGATAGAATTAAGGGAGATG	ATGGGAAAAGYGAAAATGTTAATAA
	Reverse	[Btn]ATCTACACACRCCAAAAAACTAATATCCC	
*LCCR4*	Forward	TGGAAAGAGGAGTTTTTAGTTTATTTAAG	AAATTTTAGGYGATGGTGAATTA
	Reverse	[Btn]ATTACCTACCACAAAAACTTCATAATAT	
*MAGEA2*	Forward	GATTTGYGTATTGGAGGTTAGAGGATA	GTAAGAYGTYGAGGGAGGATTGA
	Reverse	[Btn]TAAAAAATCTTCCCCTACAAAATAATCCA	
*MGMT*	Forward	TTTTTGGAGAGYGGTTGAGTTAGGT	AGGTTATYGGTGATTGTAGTT
	Reverse	[Btn]CCAAACCAACAAAAACCCTATCA	
*NNAT*	Forward	TGTAGGTTAGGGATTGGGGAGAA	TTAAAGTAAAATTTAAAAGTAAGT
	Reverse	[Btn]TCCATCTTAACCCCCTTCCAA	
*TSPAN32*	Forward	GAGGTTTATAAAGTTTTTTTTTGGAGG	GAGGTTTTAYGTGAGTGTGA
	Reverse	[Btn]CACCCTTTAAAATATCCTATAACAACTT	
*HPN*	Forward	ATGAAATAAAGATTTTTGGATTTGATGTAT	GTGAGTTTYGTTATTTTTTTTTTAT
	Reverse	[Btn]TAAATAACTTCACCTATAAACCCTCAAAT	
*JAK2*	Forward	[Btn]TTTTTTAGATAGTTATGGGATTGGTTTAT	AATAAAAACRACAAAACAACAAACA
	Reverse	ACACTCCTTACCCTACTAAATTATATT	
*PAX 8*	Forward	GTGATTTAGGAGGATTTAGAGAATTTTATT	ATTTTTTTGTATTTAGTTAGTTAA
	Reverse	[Btn]CTCTCCTCCTTCTAAAATTTATTCC	
*TDGF1*	Forward	ATTGGGGTTTGTTGTTGAAGAA	TTTATTTTTTTTTTAAATTGTTATT
	Reverse	[Btn]AAACAACCAAAAAAAAACATTCATCTCC	
*RB1*	Forward	[Btn]TTGGGGTTGGTTTATTTATTTAGTTTTG	TTACCCCTCCTCCCC
	Reverse	AACRAAAAACCCTTACCCCTCCTC	

### Statistical analysis

The methylation status at each array probe was established by analyzing the distribution of *b*-values for all samples and CpG sites and transforming data into discrete values. The cutoff values for hyper- and hypomethylation were established using the minAS method [39] and considering the bimodal distribution of *b*-values. Differential methylation analysis was done at the single CpG, CpG island, and gene-centric levels. For the analysis of CpG island, individual probes were considered independent observation, whereas the gene-level analysis simply counted the percentage of CpG and CpG island associated to each gene that were declared significant. Differential methylation across the NB subgroups was determined by either a proportion test when several groups were compared or by Fisher’s exact test when only two subgroups were involved. Subgroups were established and compared based on clinical and biological parameters such as age at first diagnosis (younger vs. older than 18 months), *MYCN* status (*MYCN*-amplified vs. non-amplified tumors), stage (L1-L2 vs. M-MS), risk groups (low and intermediate vs. high risk), relapse, and death (patients with events (relapse/death) vs. patients without events). Nominal *P* values were corrected for multiple tests using the Benjamini and Hochberg FDR procedure [40].

Data distribution from pyrosequencing analysis was not bimodal and varies among genes; therefore, variables were analyzed as continuous. The relationship between the methylation status and NB risk factors was evaluated using a MANOVA test. A Cox elastic net analysis [41] was performed to evaluate the influence on survival of gene promoter methylation. This novel statistical analysis method is especially suited for analyzing data with many variables and few observations by performing variable selection. This is done by penalizing predictors’ coefficients towards zero according to their association with survival. Coefficients from variables with less influence on survival were more penalized, dropped to zero, and excluded from the predictive model.

For all the above-mentioned statistical tests, R software (version 3.0.2) and package glmnet (version 1.9-5) were used. For EFS analysis, time to event was defined as the time from diagnosis until the time of first occurrence of relapse, progression, or death. For OS, time to event was defined as time until death or until last contact if the patient was alive. *P* values <0.05 were considered statistically significant.

## Additional files

Additional file 1:
**Schematic mapping of CpGs sites within the promoter regions of the sixteen genes selected for pyrosequencing validation.**
Additional file 2:
**Illumina methylation microarray data of the sixteen genes selected for bisulfite pyrosequencing validation.**
Additional file 3:
***TDGF1*** **and** ***RB1*** **methylation data from bisulfite pyrosequencing analysis in the validation cohort of patients.**
Additional file 4:
**Correlation graphs between** ***TDGF1*** **expression and** ***DNMT1*** **(A),** ***DNMT3B*** **(B) and** ***DNMT3A*** **(C) obtained at R2: microarray analysis and visualization platform (** http://r2.amc.nl **) using a cohort of 31 NB patients with** ***MYCN*** **amplification.**
