# Supplementary figures and images for: Two independent epigenetic biomarkers predict survival in neuroblastoma
Source: Clin Epigenetics. 2015 Feb 27;7(1):16. doi: 10.1186/s13148-015-0054-8 (PMC4357365; doi:10.1186/s13148-015-0054-8)

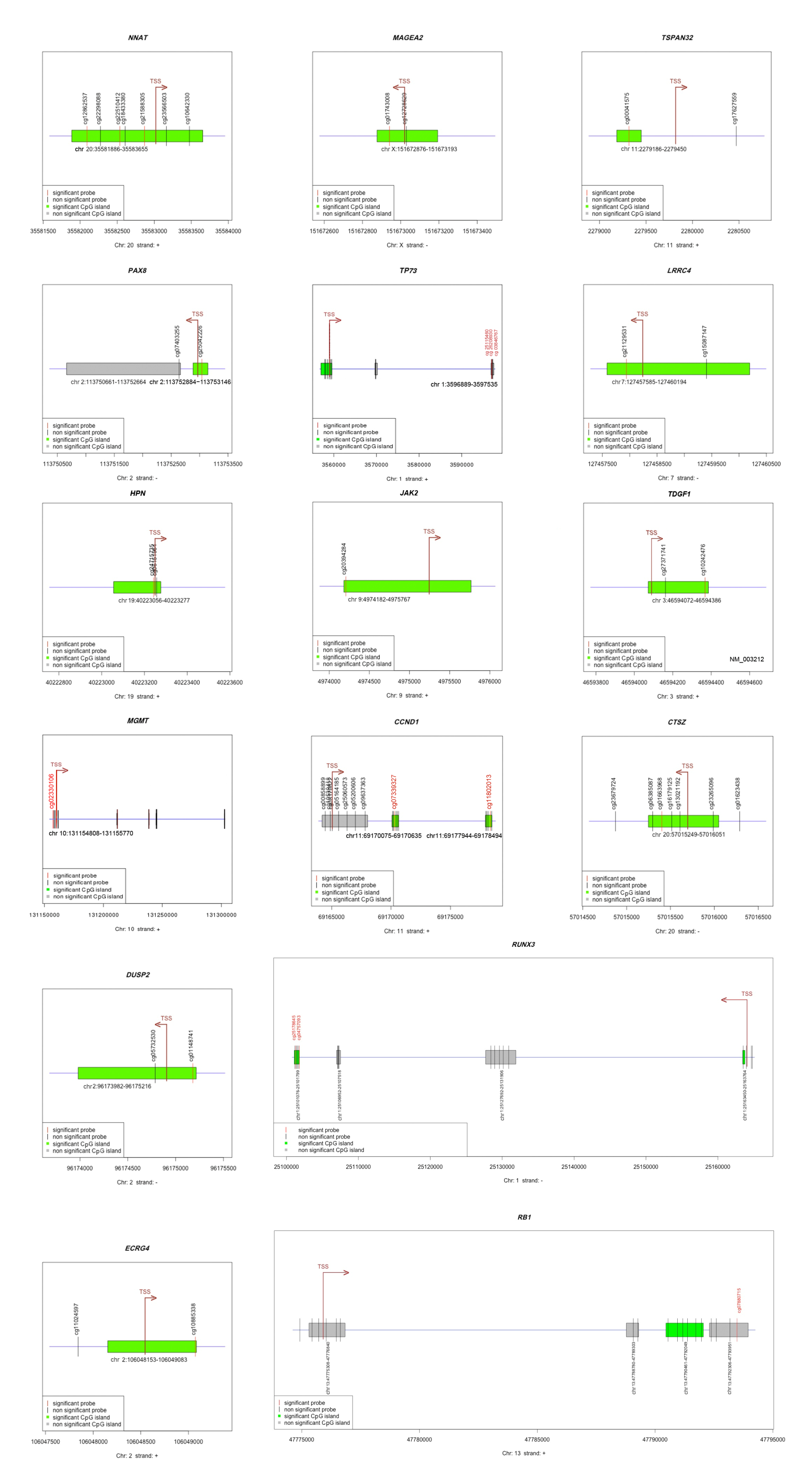

Supplement: Additional file 1: — Schematic mapping of CpGs sites within the promoter regions of the sixteen genes selected for pyrosequencing validation. [file 13148_2015_54_MOESM1_ESM.tiff]

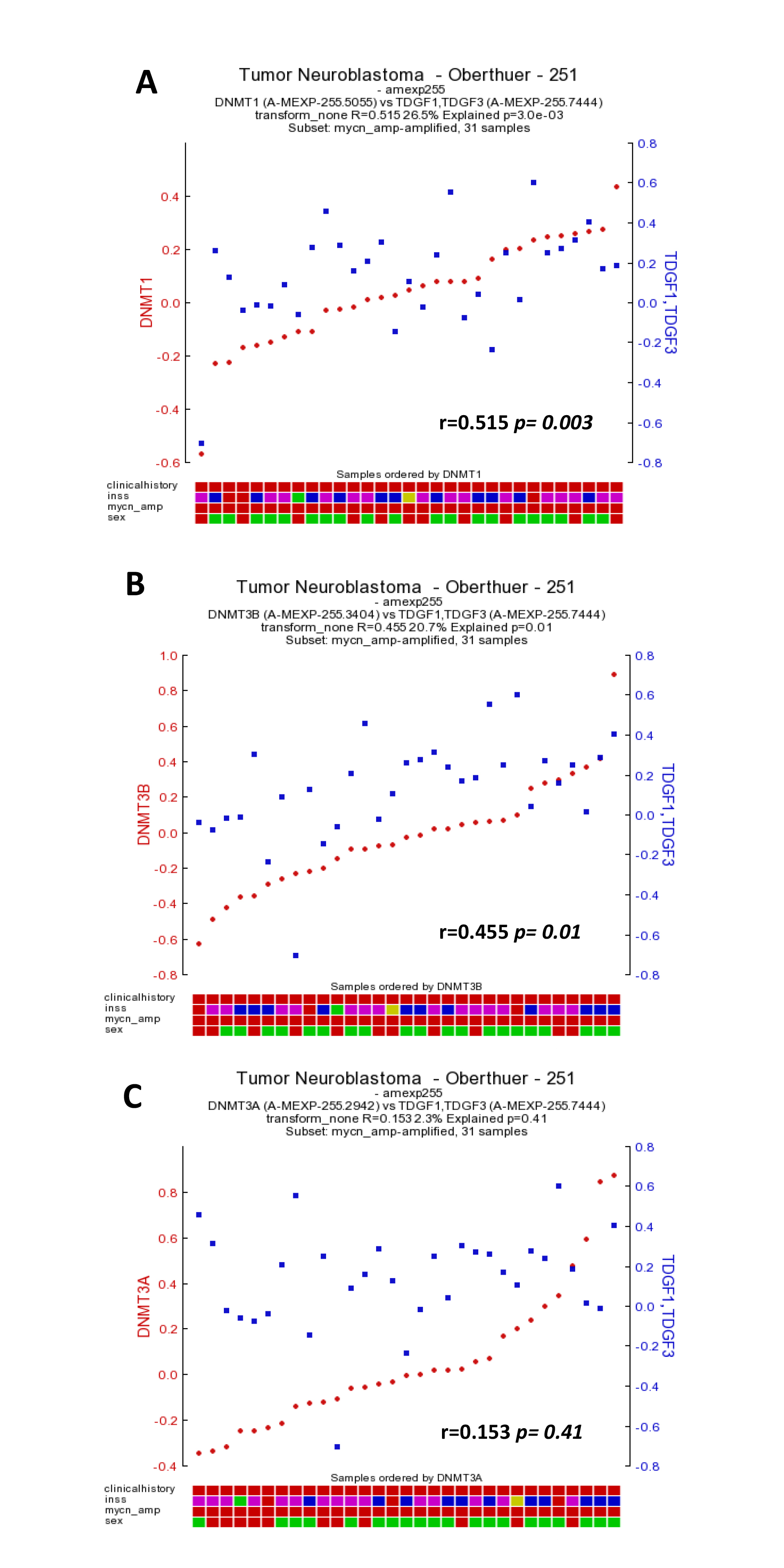

Supplement: Additional file 4: — Correlation graphs between TDGF1 expression and DNMT1 (A), DNMT3B (B) and DNMT3A (C) obtained at R2: microarray analysis and visualization platform ( http://r2.amc.nl ) using a cohort of 31 NB patients with MYCN amplification. [file 13148_2015_54_MOESM4_ESM.jpg]
